# Length and associated characteristics of short-term detentions: an analysis of detentions under the Mental Health Act in Scotland, 2006–2018

**DOI:** 10.1007/s00127-023-02459-3

**Published:** 2023-03-30

**Authors:** Moira Connolly, Lisa Schölin, Gail S. Robertson, Arun Chopra

**Affiliations:** 1 Mental Welfare Commission for Scotland, Edinburgh, Scotland; 2 Centre for Cardiovascular Science, University of Edinburgh, Edinburgh, United Kingdom; 3 School of Mathematics, University of Edinburgh, Edinburgh, Scotland

**Keywords:** Mental Health Act, Involuntary admission, Detention, Scotland, Mental health services, Short-term detention certificates

## Abstract

**Purpose:**

The Mental Health Act in Scotland is under review. Previous iterations increased patients’ rights but the maximum time for short-term detentions remains unchanged, despite evolving psychiatric treatment models. We explored length, mode of ending and factors of influence on the application of short-term detention certificates (STDCs), which can last up to 28 days, across Scotland between 2006 and 2018.

**Methods:**

Data on age, gender, ethnicity, date of commencement and ending of the STDC and detention site from all 42,493 STDCs issued to 30,464 patients over 12 years were extracted from the national repository for detentions under the Mental Health (Care and Treatment) (Scotland) Act 2003 and analysed using mixed models.

**Results:**

One in five STDCs lapsed on day 28. Two in five were revoked and the remainder extended to a treatment order. STDCs that were not extended averaged 19 days, and revoked STDCs 14 days. The probability of a detention lapsing varied across hospitals and increased with patient age. The odds of a detention lapsing on day 28 were 62% lower and revoked detentions 10% shorter in 2018 relative to 2006. The odds of a detention extending decreased significantly from 2012 to 2018. Extended STDCs were associated with increased patient age, male gender, and ethnicity other than White Scottish. There was little initiation of or active revocation of STDCs on weekend days.

**Conclusion:**

The length of STDCs reduced over time, fewer detentions lapsed, and weekday patterning was evident in each year. These data can inform legislative and service reviews.

**Supplementary Information:**

The online version contains supplementary material available at 10.1007/s00127-023-02459-3.

## Introduction

The Mental Health (Care and Treatment) (Scotland) Act 2003 (‘the 2003 Act’) is being reviewed by the Scottish Mental Health Law Review (SMHLR), with their consultation phase concluded by September 2022 [1]. Following the 2001 ‘Millan Review’ of the Mental Health (Scotland) Act 1984 (‘the 1984 Act’), sweeping changes of functions were made. This included the adoption of a set of principles to underpin human rights law, the setting up of a new Mental Health Tribunal to determine rights in a judicial capacity, the introduction of the term ‘mental disorder’ to refer to mental illness, personality disorder and learning disability and enabling compulsory treatment in the community. Of note, no changes were made to the existing periods during which emergency detention^1^ and short-term detention of a patient could occur [2]. However, at that time there were no data available on whether short-term detentions were lasting longer than necessary, and no changes were made leaving the maximum detention time for assessment and treatment the same as in legislation set out in 1984, i.e., 28 days [3].

Section 49 of the current 2003 Act, sets out a duty for the Responsible Medical Officer (RMO) to review the detention regularly. The still current SMHLR review is an opportunity to consider whether these timeframes remain appropriate given the swathe of new treatments and service delivery models in the intervening decades and with the added requirements of international treaties and jurisprudence in mental health law. The SMHLR’s terms of reference explicitly refer to the United Nations Convention on the Rights of Persons with Disabilities (UNCRPD) [1]. One of the requirements of Article 12(3) of the UNCRPD is that safeguards are in place that ensure that measures relating to the exercise of rights, will and preference of the individual are proportional and apply for the shortest period of time possible [4].

Length of detentions was a focus for the 2018 Independent Review of mental health legislation in England and Wales [5]. One concern was that some detentions were lasting longer than necessary, leading to recommendations on developing a care and treatment plan reviewed by the clinical director within 14 days of a detention under 'Section 2' (the nearest equivalent to the short-term detention certificate (STDC) in Scotland) [5]. In Scotland, admissions and length of stay (LOS) in psychiatric inpatient settings have decreased over time due, in part, to an increase in community-based care and treatment [6]. Between 2010 and 2020 the proportionate spend on community mental health rose from 26% to 32%, data for individual health boards shows that the degree of proportionate change varied.^2^ In parallel, rates of detention have been increasing in many countries [7] and in Scotland there has been a gradual increase, year-on-year, of the numbers of people who experience mental health treatment under detention over the last decade [8].

The relationship between detention and hospital LOS is complex and inconsistent. A study in one English inner-city trust found detained patients to have longer LOS [9] whereas a systematic review of evidence from the US reported that being subject to mental health legislation was associated with shorter LOS [10]. Cross-country comparisons have been made of differences in the structural timings and procedural stages of detention [7], but patient and service-related factors, which might influence the length of initial assessment and treatment orders in clinical practice, have received less attention.

To date there has been no in-depth study exploring how long STDCs last. The aim of this study was to explore their length, the influence of patient characteristics such as age, sex and ethnicity, admission site, changes over time, and if there were differences in use across the week. We addressed four specific questions:

1. What is the average length of a STDC? Does age, gender or ethnicity influence duration of revoked STDCs, or the proportion of STDCs that are lapsed or extended?
2. Does duration of revoked STDCs or proportion of STDCs that are lapsed or extended vary between the busier (by numbers) admission sites and is age an influencing factor?
3. Has the length of STDCs changed since 2006? Does age, gender or ethnicity influence changes over time in duration of revoked STDCs, or the proportion of STDCs that are lapsed or extended?
4. For revoked and lapsed STDCs, is there a difference across the days of the week as to when detentions are initiated, revoked, or lapsed?

## Methods

### Study population and data source

The Mental Welfare Commission for Scotland (‘the Commission’) is notified of all detentions under the 2003 Act. We extracted Commission data on STDCs, the preferred ‘gateway order’ to compulsory powers under the 2003 Act (p.14) [11]. STDCs offer an extensive set of rights to the patient and their named person. This differs from the emergency detention certificate, which has no right of challenge in its 72-h period [11]. This study includes information relating to the detention, which is reported to the Commission, but does not pertain to any period of the admission that was on a voluntary basis.

All patients with a record of one or more STDCs under 'Section 44' of the 2003 Act between 1 January 2006 and 31 December 2018 were included and all incident detentions followed to conclusion of the STDC. We coded each patient to enable identification of multiple STDCs for individuals. The admission sites were composed of all types of hospitals while the in-depth site-based analysis (research question 2) included the nine most common sites for detention, which are all psychiatric hospitals or mental health wards in general hospitals.

The Commission is a national body with statutory responsibility for monitoring and reporting on use of mental health and incapacity legislation. When performing these duties the Commission does not rely on individuals’ consent. The legal ground for processing this personal data is the substantial public interest: Article 6 (1) (e) and 9 (2) (g) of the General Data Protection Regulation (GDPR) and Data Protection Act 2018, Schedule 1 paragraph 6, “for the exercise of a function conferred on a person by enactment or rule of law”.

This study analysed only anonymised, secondary data and followed standard protocols for processing personal data. It was overseen by the Commission’s Caldicott guardian and Information Governance Manager. The Commission’s privacy notice is available on its website [12]. The data that support the study findings are available from the Commission, with restrictions.

### Data

**Fig. 1:**
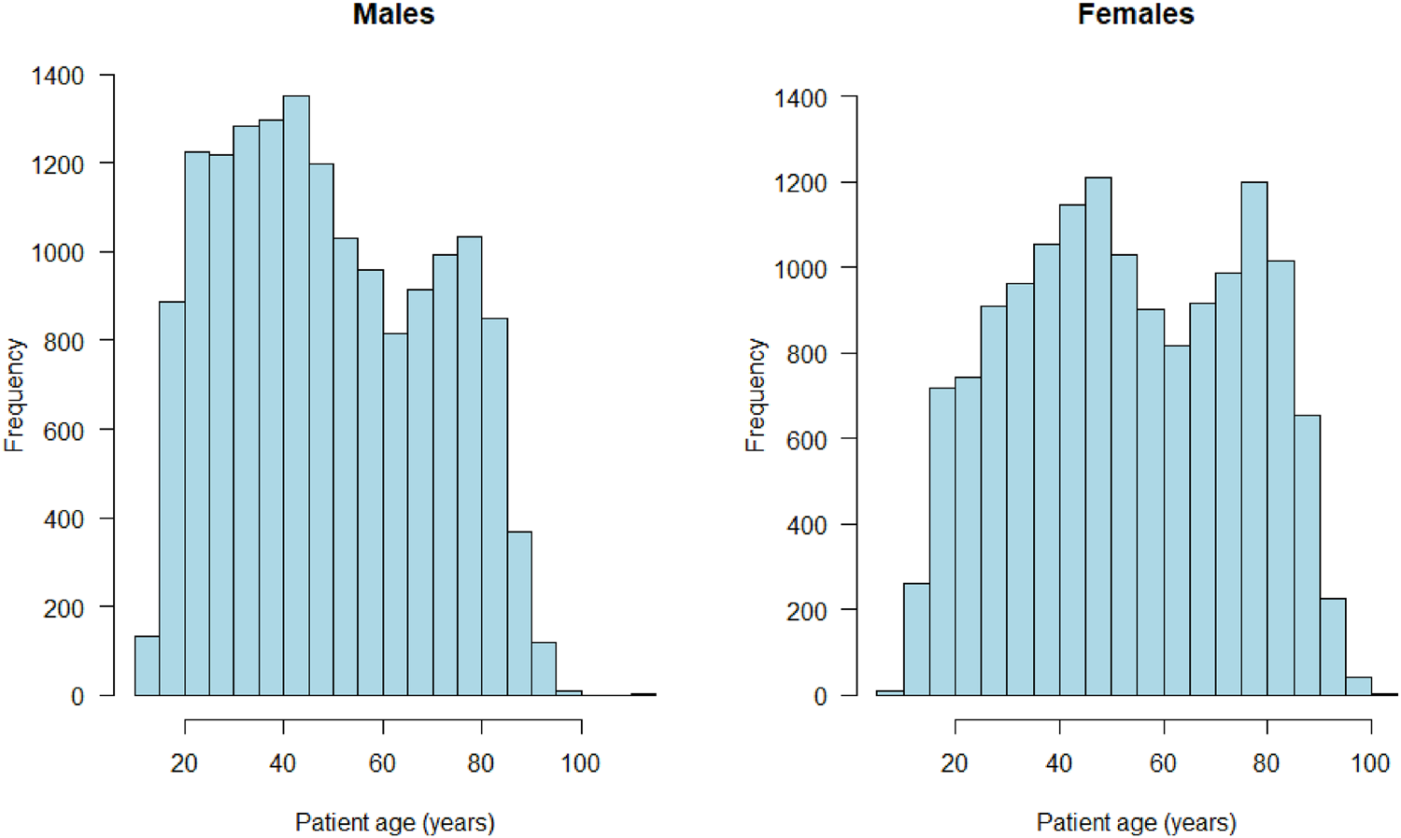
Histograms showing distribution of STDC patient age for **a** male (*n* = 15,674) and **b** female patients (*n* = 14,790) illustrating bimodal distribution

For each detention, information was extracted on the patient’s age, gender and ethnicity, start and end date of the STDC and the admission site, including all public and private hospital sites. Data on ICD-10 diagnosis were not included, as this information is not consistently retrievable from the information management system. Figure 1 shows the age distribution of detained patients by gender with a bimodal distribution in ages of both male and female patients and peak numbers of patients detained in their 40s and 80s. Ethnicity was categorised into census categories: ‘White Scottish’, ‘White Other British’, ‘White Other’, ‘Asian’, ‘African, Caribbean or Black’, ‘Mixed’, and ‘Other’. We explored three different STDC outcomes:

- Certificates revoked before the final day (day 28) of the detention (hereafter ‘revoked’). This requires action by a psychiatrist in charge of the patient’s care, (or another formalised ending such as a successful appeal, leaving Scotland, or death).
- Certificates that ended on day 28 (hereafter ‘lapsed’). This does not require action from the psychiatrist.
- Certificates that lasted the full 28 days and detention extended, most often as a Compulsory Treatment Order (CTO), following a Tribunal hearing, initially for up to six months (hereafter ‘extended’). The duration of detentions extended beyond STDC was not explored and ‘extended’ STDCs are not included in estimates of mean and median STDC duration.

**Table 1:** Descriptive statistics for detentions contributing to statistical analyses

Individual-level variables	Revoked (*N* = 16,610)	Lapsed (*N* = 9393)	Extended (*N* = 16,490)
Age groups (mean age ± sd, median)			
< 18 years	15.7 ± 1.4 (16)	15.6 ± 1.4 (16)	15.4 ± 1.5 (16)
18–65 years	40.1 ± 12.6 (40)	42.7 ± 12.9 (43)	41.1 ± 13.1 (41)
> 65 years	77.5 ± 7.4 (77)	77.7 ± 7.2 (77)	76.8 ± 6.8 (77)
Age groups (*n*, %)			
< 18 years	522 (3.1)	152 (1.6)	543 (3.3)
18–65 years	12,857 (77.4)	5916 (63.0)	11,410 (69.2)
> 65 years	3231 (19.5)	3325 (35.4)	4537 (27.5)
Gender (*n*, %)			
Female	8376 (50.4)	5026 (53.5)	7905 (47.9)
Male	8234 (49.6)	4367 (46.5)	8585 (52.1)
Ethnicity^a,b^ (*n*, %)			
African, Caribbean or Black	118 (1.6)	36 (0.9)	157 (1.5)
Asian	171 (2.3)	92 (2.4)	302 (2.8)
Mixed	16 (0.2)	11 (0.3)	75 (0.7)
Other	36 (0.5)	19 (0.5)	33 (0.3)
White: Scottish	6174 (83.4)	3376 (86.9)	8636 (81.4)
White: Other British	499 (6.7)	207 (5.3)	845 (8.0)
White: Other	391 (5.3)	143 (3.7)	561 (5.3)

^a^The ethnicity dataset comprised of a total of 21,898 STDCs (revoked *n* = 7405; lapsed *n* = 3884; extended *n* = 10,609)

^b^Scotland’s population was 96% White, 2.7% Asian, 1% African, Caribbean or Black and 1% Mixed or Other Ethnic group according to the 2011 census Ethnicity | Scotland's Census (scotlandscensus.gov.uk) accessed 19 November 2022

We used different datasets to address the study questions. We merged our primary dataset for STDCs with a dataset specifying the ethnicity of individuals detained under an STDC. The primary dataset contained 42,493 STDC incidents relating to 30,464 patients admitted to 189 admission sites.

Summary statistics on patient age group, gender, and ethnicity (excluding patients with no information on ethnicity) for each STDC group (revoked, lapsed, extended) are reported in Table 1, and age distribution by gender in Fig. 1. The dataset relating only to revoked and lapsed STDCs totalled 26,003 STDCs and the dataset which also included those which were extended consisted of a total of 42,493 STDCs. For the dataset containing detention sites in which each had at least 900 STDCs in the period studied (*N* = 8) the dataset comprised 18,382 STDCs.

### Analysis

To address Question 1, we calculated summary statistics showing mean and median length of stay for revoked STDCs and proportions of total STDCs that lapsed or were extended. A binomial generalised linear mixed model (GLMM) was used to determine the association of age, gender or ethnicity with STDCs finishing as lapsed, or being extended, and a Poisson-distributed GLMM for associations with length of revoked STDCs. To address Questions 2 and 3 we used three statistical models. A binomial generalised linear mixed model (GLMM) examined which demographic variables may explain whether a STDC lapsed. A GLMM with a Poisson distribution examined which demographic variables may explain duration of STDCs that were revoked before day 28. A binomial GLMM determined which explanatory variables affected whether an STDC was extended beyond 28 days. All models accounted for variation in duration of detention of patients admitted to different hospitals and for variation in detention lengths among patients admitted multiple times by including patient ID and hospital as intercept only random effects in models. Year was also included in models to address Question 3.

Complete information for age, gender and ethnicity was available for 21,898 detentions (14,039 patients from 161 admission sites), as 20,595 STDCs lacked ethnicity information. No data were missing for age or gender. This dataset was used to address both Questions 1 and 2, although subsets of the dataset including revoked and lapsed (*n* = 11,289) and revoked only (*n* = 7405) STDCs were used for analyses examining STDCs <  = 28 days in length. The binomial GLMMs included age, gender, ethnicity and year as explanatory variables, and patient and hospital as random effects (after selecting the best random effects structure using Akaike’s Information Criteria (AIC) and number of degree of freedom of models with different random effects). The model’s goodness-of-fit was assessed by calculating the area under a receiver operating characteristic curve (ROC curve). The Poisson-distributed GLMM included the same explanatory variables and random effects and was checked for overdispersion. Only STDCs that included data for all these explanatory variables were included in models. After selecting the most appropriate random effects structure for each model, the importance of each fixed effect to model parsimony was determined using an ANOVA-based model selection procedure to assess the importance of individual explanatory variables in explaining variation in each response variable (using the ‘Anova’ function from the ‘car’ package in R v3.6.1) [13].

To address the question of whether STDC duration varied by hospital and age, we examined how the duration of STDCs that were revoked varied among hospitals and ages, whether an STDC lapsed varied among hospitals and patients of different ages, and whether an STDC being extended beyond 28 days varied depending on hospital and age using three separate statistical models. A binomial generalised linear mixed model (GLMM) was used to examine which variables affected whether a STDC ended on day 28 (*n* = 10,972). A GLMM with a Poisson distribution examined which variables explained duration of STDCs of less than 28 days (*n* = 6863). Finally, a binomial GLMM was used to determine whether age and hospital affected whether an STDC was extended beyond 28 days (*n* = 18,382). All models accounted for variation among patients admitted multiple times by including patient as a random effect. An interaction between age and hospital was included in each model to determine whether the relationship between age and the response variable varied among hospitals. Comparisons between hospitals were made relative to Hospital A which had the largest number of STDCs of the eight with greatest numbers in our analysis.

To address Question 4 we examined whether STDCs were more likely to start or end on a particular day of the week (Sunday to Saturday), and we did this for i) STDCs which lapsed (*n* = 9393) and ii) STDCs which were revoked (*n* = 16,610). For both analyses, we calculated total numbers of detentions that ended on each day of the week. We used Chi-squared tests to determine whether there was a significant difference in number of STDCs that started or ended on different days of the week.

Although there may have been important interactions between some of the explanatory variables we do not consider interactions in our models as they are not relevant to our original research questions.

## Results

### Average length of STDCs and influence of age, gender, or ethnicity on duration of revoked STDCs, or the proportion of STDCs that lapsed or were extended (Q1)

Overall, 39% of STDCs were revoked before day 28, 22% lapsed on day 28 and 39% were extended to another section of the 2003 Act. The mean duration of lapsed and revoked STDCs combined was 19.33 ± 8.71 days (median = 22 days) while revoked STDCs lasted on average 14.43 ± 7.23 days (median = 14 days).

Table 1 describes the differences according to demographic characteristics. The mean and median length of revoked STDCs was highest in the age group > 65 years. Lapsed STDCs had the highest proportion of patients aged 65 or older. Within each of the age groups, the proportion lapsed were 12.5% for < 18, 19.6% for 18–65, and 30.0% in the over 65s. While the gender distribution of revoked STDCs was equal, there was a higher percentage of females among lapsed and a higher percentage of males among extended STDCs. The distribution of ethnicity was relatively similar, with lapsed STDCs having the highest proportion of White Scottish.

We found that 3884 (34%) of the 11,289 STDCs in the appropriate dataset (see Table 1 footnote) lapsed on day 28. Age was positively associated with STDCs lapsing, and with longer length of revoked STDCs. There was no effect of gender, but compared with White Scottish patients, patients of other ethnicities showed significant variation in the probability of STDCs lapsing. Age, male gender and non-White Scottish ethnicity were associated with STDCs being extended (Supplementary tables 1– 3).

### Variations across hospitals (Q2)

Across the nine admission sites with the highest numbers of STDCs (*n* = 18,382), 6863 (37%) were revoked 4109 (23%) lapsed and 7410 (40%) were extended. The probability of a detention lapsing on day 28 varied across the hospitals and increased with age (Table 2). The interaction between age and hospital was marginally significant at a p-value of 0.04.

**Table 2:** Probability of STDCs lapsing depending on age and hospital

	Coefficient	Std error	z-value	*p*-value	Wald chi-square	Df	*p*-value
Hospital:	–	–	–	–	88.78	7	< 0.001
B	1.21	0.30	4.03	< 0.001	–	–	–
C	0.33	0.22	1.50	0.13	–	–	–
D	0.17	0.21	0.78	0.43	–	–	–
E	0.78	0.24	3.30	< 0.001	–	–	–
F	0.50	0.26	1.93	0.05	–	–	–
G	– 0.01	0.26	– 0.03	0.97	–	–	–
H	0.77	0.19	4.03	< 0.001	–	–	–
Age	2.35	0.27	8.64	< 0.001	326.20	1	< 0.001
Age: Hospital	–	–	–	–	15.02	7	0.04
Age: B	– 1.26	0.71	– 1.78	0.08	–	–	–
Age: C	0.34	0.43	0.79	0.43	–	–	–
Age: D	0.21	0.41	0.52	0.60	–	–	–
Age: E	– 0.63	0.47	– 1.32	0.19	–	–	–
Age: F	– 0.04	0.45	– 0.09	0.93	–	–	–
Age: G	0.71	0.50	1.44	0.15	–	–	–
Age: H	– 0.58	0.38	– 1.54	0.12	–	–	–

*N* = 10,972, AUC = 0.81; The random effect included in the model was patient (*n* = 8809). Note that age was rescaled by dividing values by 100. Model output is displayed (coefficient estimates, standard error around estimates, z-values and p-values associated with each variable level) and coefficient values and standard errors are given in respect to Hospital A. Values of Wald Chi-square tests comparing models including and excluding each variable and associated p-values are also displayed

We found that the duration of revoked STDCs increased significantly with patient age and varied significantly across hospitals. Age had a varying effect on duration of STDCs and this effect varied among hospitals (Table 3).

**Table 3:** Duration of revoked STDCs by age and hospital

	Coefficient	Std error	z–value	*p*-value	Wald chi-square	df	*p*-value
Hospital:	–	–	–	–	41.87	7	< 0.001
B	– 0.02	0.09	– 0.20	0.84	–	–	–
C	0.23	0.06	3.83	< 0.001	–	–	–
D	0.22	0.06	3.87	< 0.001	–	–	–
E	0.16	0.06	2.63	0.009	–	–	–
F	0.16	0.07	2.28	0.02	–	–	–
G	0.09	0.07	1.36	0.17	–	–	–
H	0.08	0.05	1.53	0.13	–	–	–
Age	0.70	0.08	9.24	< 0.001	206.54	1	< 0.001
Age: Hospital	–	–	–	–	14.74	7	0.04
Age: B	0.19	0.21	0.89	0.37	–	–	–
Age: C	– 0.27	0.13	– 2.12	0.03	–	–	–
Age: D	– 0.33	0.12	– 2.81	0.005	–	–	–
Age: E	– 0.22	0.14	– 1.65	0.10	–	–	–
Age: F	– 0.30	0.13	– 2.20	0.03	–	–	–
Age: G	– 0.08	0.15	– 0.52	0.61	–	–	–
Age: H	– 0.21	0.11	– 1.93	0.05	–	–	–

*N* = 6,863; The random effect included in the model was patient (*n* = 5772). Note that age was rescaled by dividing values by 100. Model output is displayed (coefficient estimates, standard error around estimates, *z*-values and *p*-values associated with each variable level) and coefficient values and standard errors are given in respect to Hospital A. Values of Wald Chi-square tests comparing models including and excluding each variable and associated *p*-values are also displayed

Finally, we examined the effect of age and hospital on whether STDCs were extended. The probability varied across hospitals and increased significantly with patient age (Table 4). There was a significant interaction between age and hospital, suggesting that the relationship between age and whether a STDC was extended varied across hospitals (Supplementary table 3) (Table 5).

**Table 4:** Probability of STDCs being extended depending on age and hospital

	Coefficient	Std error	z-value	*p*-value	Wald chi-square	df	*p*-value
Hospital:	–	–	–	–	40.18	7	< 0.001
B	0.04	0.22	0.19	0.85	–	–	–
C	0.19	0.16	1.22	0.22	–	–	–
D	– 0.48	0.15	– 3.17	0.002	–	–	–
E	0.07	0.17	0.40	0.69	–	–	–
F	0.41	0.19	2.11	0.03	–	–	–
G	– 0.04	0.17	– 0.21	0.83	–	–	–
H	0.05	0.14	0.35	0.68	–	–	–
Age	0.43	0.19	2.26	0.02	11.81	1	< 0.001
Age: Hospital	–	–	–	–	40.18	7	< 0.001
Age: B	0.14	0.52	0.27	0.79	–	–	–
Age: C	– 0.61	0.31	– 1.94	0.05	–	–	–
Age: D	0.79	0.29	2.69	0.007	–	–	–
Age: E	– 0.48	0.36	– 1.33	0.18	–	–	–
Age: F	– 1.14	0.34	– 3.31	< 0.001	–	–	–
Age: G	0.39	0.34	1.15	0.25	–	–	–
Age: H	– 0.37	0.28	– 1.34	0.18	–	–	–

*N* = 18,382, AUC = 0.90; The random effect included in the model was patient (*n* = 13,335); Note that age was rescaled by dividing values by 100. Model output is displayed (coefficient estimates, standard error around estimates, *z*-values and *p*-values associated with each variable level) and coefficient values and standard errors are given in respect to Hospital A. Values of Wald Chi-square tests comparing models including and excluding each variable and associated p-values are also displayed

**Table 5:** Numbers and proportions of STDCs starting and ending on each day of the week

		Day of the week *n* (%)						
		Mon	Tues	Wed	Thu	Fri	Sat	Sun
Started	Lapsed^a^	1481 (15.8)	1639 (17.4)	1624 (17.3)	1737 (18.5)	2191 (23.3)	408 (4.3)	313 (3.3)
	Revoked^b^	2762 (16.6)	2497 (15.0)	2640 (15.9)	2893 (17.4)	3621 (21.8)	1138 (6.9)	1059 (6.4)
Ended	Lapsed^c^	1639 (17.4)	1624 (17.3)	1737 (18.5)	2191 (23.3)	408 (4.3)	313 (3.3)	1481 (15.8)
	Revoked^d^	4218 (25.4)	3389 (20.4)	3131 (18.9)	2758 (16.6)	2855 (17.2)	151 (0.9)	108 (0.7)

^a^*Χ*^2^_6_ = 2232, *p* < 0.001, *n* = 9393; ^b^*Χ*^2^_6_ = 2362.7, *p* < 0.001, *n* = 16,610; ^c^*Χ*^2^_6_ = 2232, *p* < 0.001, *n* = 9393; ^d^*Χ*^2^_6_ = 6514.9, *p* < 0.001, *n* = 16,610

### Changes in length of STDCs over time (Q3)

The duration of revoked and lapsed STDCs, combined, declined over time, with less of a decline in median duration of revoked STDCs (Supplementary Fig. 1). The proportion of STDCs that lapsed decreased over time but there was little change to the proportion extended (Supplementary Fig. 2). The probability of STDCs lapsing changed significantly after the first reporting year (2006), and increased significantly with patient age. The coefficients estimated from the model show that relative to 2006, the odds of a detention lapsing was 62% lower in 2018. The length of revoked STDCs declined significantly over the years but increased significantly with patient age. Model coefficients show that in comparison with 2006, the duration of revoked STDCs decreased by more than 10% in 2018 (Supplementary table 2).

Model results show that the probability of a STDC being extended changed significantly from 2006 and increased significantly with patient age. Relative to 2006, the odds of a detention being extended decreased significantly from 2012 to 2018. STDCs were extended beyond 28 days as patient age increased and for male patients (Supplementary table 3).

### Day of the week orders started and ended (Q4)

For both lapsed and revoked STDCs, there was significant variation in the day of the week orders began and ended. On weekdays the number of STDCs started which ended as lapsed or revoked ranged from 4136 (Tuesday) to 5812 (Friday), dropping at the weekends to 1546 on Saturdays and 1372 on Sundays making weekends the least active days for revocations.

More than three times as many lapsed STDCs ended on a Sunday as on a Friday or Saturday, reflecting that these will have been initiated on a Monday. Table 5 shows the number of STDCs which started/ended on each day (with results of Chi-square tests carried out on day of the week included as footnotes).

## Discussion

We have demonstrated that between 2006 and 2018 for every five people detained, two had their STDC revoked, one had theirs lapse and two extended beyond 28 days, and by 2018 the average length of STDCs not further extended was 19.3 days. Relevant explanatory factors may include those already known to influence detention, and LOS. A systematic review, meta-analysis, and narrative synthesis of clinical and social factors found a diagnosis of psychotic or bipolar disorder and previous detention increased the odds of detentions [14]. Other factors included male gender, unemployment, receiving welfare benefits, being unmarried, and living in an economically deprived area [14]. Our analysis extended findings on male gender to include an association with longer mean duration for STDCs and an increased likelihood of STDCs being extended.

Evidence from a systematic review and meta-analysis of international data on ethnic variations in detention suggests people from Black Caribbean, Black African, and south Asian ethnic groups, and migrants, are more likely to experience detention compared to white ethnic groups [15]. Explanations for increased LOS among detained patients from black ethnic background included increased prevalence of psychosis, increased perception of risk of violence, increased police presence, absence of or mistrust of GPs, and ethnic disadvantages [15, 16]. Our finding that once detained under an STDC, patients from ethnic backgrounds other than White Scottish are more likely to have their detention extended, is new. This may be explained by differences in how risk is perceived. Recent work from the Commission [17] has shown that black people and mixed race people are more often recorded as of greater risk to ‘self and others’ than to themselves, compared to all other ethnicities where people are more often recorded as a risk to self rather than to ‘self and others’. This difference [17] was more marked for black women in comparison to white.

Our finding that older age was associated with longer and extended STDCs (more so over time) is consistent with 2019– 20 mental health act statistics for England, which showed greater detention rates and longer 'Section 2'  detentions in over 65s compared to younger adult groups [18]. This may reflect greater use of the Acts in an age group in which impaired capacity and higher rates of confusion predominate [19, 20]. This has implications for mental health service planning in Scotland [21], where the number of over 65s in the population increased by 37% over a 30-year period while 0–15-year-olds fell by 10% [22], requiring adequate provision of age appropriate inpatient services.

The analysis by admission site revealed consistency in the impact of age on the proportion of revoked, duration, and extension of STDCs; however, the relationship between age and probability of extension varied among hospitals. There may have been confounders relating to the hospitals—e.g., they may vary in their rural/urban setting and inpatient and community service provision—or patient population, in keeping with research showing that rates of detention vary significantly between local areas and services and seem to reflect local factors, especially socioeconomic and ethnic population composition [25].

Our study suggests evolving practice. By 2018 the mean duration of revoked STDCS was 14 days, and for all STDCs 19.3 (median 22 days), similar to experimental data from England in 2019–20 where the mean and median was 19 days for detentions under 'Section 2'  [18]. Our finding that fewer detentions lapsed in 2018 compared to 2006, may reflect a continuing trend first described with the 1984 Act in South Glasgow when around half of  'Section 26' detentions (STDC equivalent) lapsed at day 28 [24]. The observed decrease in the average duration of revoked STDCs may signify increasing familiarity with the 2003 Act’s application, akin to differences seen between clinicians of different grades [19]. It might also suggest that inpatient assessment is increasingly achieved in shorter timeframes, reflective of reduced LOS [6],

The disproportionate lapsing of STDCs warrants further attention. In 2018 the disproportionate patterning by the duration available until a safeguard kicks in, seen with 'Section 3  Detention for Treatment' orders in England and Wales, was viewed as inappropriate, ‘reflexive’, and a focus for possible reform [5]. The act of ending a patient’s period of detention under the STDC should be as active an undertaking as extending detention. Where continued detention is indicated, sufficient time must be given to prepare tribunal applications to enable patients and representatives to prepare their case. In the Commission’s most recent Mental Health Act monitoring report, 29% of all detentions, which progressed to CTO from STDC, were bridged by an interim CTO (iCTO) [8] (used when detention criteria are met but the Tribunal lacks needed information to make a full order)[11]. If late decisions to apply to the Tribunal are a factor in the granting of iCTOs then by highlighting the need for timely review there is the potential to limit reliance on interim orders and 'Section 47' extension certificates.

Lastly, we have demonstrated that the five-day working week impacts on use of the 2003 Act, with limited application of or active revocation of STDCs at the weekend. Of STDCs that lapsed, similar numbers lapsed on Sundays as on Mondays to Thursdays, the STDC having been most commonly initiated on a Monday. It is possible that patients who did not remain voluntarily were discharged on Sundays, with fewer community supports. Using the available 28 days to deliver treatment under the 2003 Act will have served individual patients well but without knowing what happened next we are left uncertain as to whether earlier revocation or continued detention might have been the better outcome.

### Implications for a new act

Despite noting the “lack of statistics on the average length of such detentions (a Section 26 order)” p.100), the Millan Review concluded that maintaining a 28-day timescale would optimise treatment planning ahead of applying for longer term detention. However, from the evidence we present the continued appropriateness of 28 days is questionable and greater focus is needed on how STDCs end. Of note, the suggestion of a clinical director-led or delegated review by day 14 in England [5] received lukewarm responses [25].

One approach might be to introduce an earlier formal review point by the RMO to either apply to the tribunal for a CTO (from which point the STDC continues) or revoke the STDC. Doing so by the end of week 3 (just over the mean length of non-extended STDCs) could be considered.

### Strengths and limitations

This study used a national dataset, with the number of STDCs available for analysis of sufficient power to detect the impact of detention indicators [10], however, there were gaps in ethnicity data for almost half the records and the small number of non-White Scottish patients may limit our ability to draw strong conclusions based on ethnicity. Additional limitations are the absence of diagnosis, and the impact of level of deprivation using postcode data. Gender existed only as binary male/female. We have reported significance at the 0.05 level, whereas with the multiple factors used in our analysis across sites a significance level of 0.01 might be preferable.

Despite limitations, our work brings some degree of “fine grained research”[14] into the processes which surround and potentially influence decision-making about detention and signals the potential for using routinely collected Mental Health Act data for research, and legislative reform. Furthermore, we support the call for integrative research including qualitative studies of clinical decision-making processes and patient experiences of detention [14].

## Supplementary Information

Below is the link to the electronic supplementary material.

Supplementary file1 (DOCX 3507 kb)

## Data Availability

The data that support the findings of this study are available from the Commission and are not publicly available due to restrictions on access.

## Footnotes

1 In this article a detention refers to an involuntary admission under statutory provisions for treatment of a mental disorder.

2 Excludes child and adolescent services. Personal communication Public Health Scotland, October 2022.
