# Supplementary material for: Length and associated characteristics of short-term detentions: an analysis of detentions under the Mental Health Act in Scotland, 2006–2018
Source: Soc Psychiatry Psychiatr Epidemiol. 2023 Mar 30;58(9):1343–52. doi: 10.1007/s00127-023-02459-3 (PMC10423135; doi:10.1007/s00127-023-02459-3)
Supplement: Supplementary file 1 — Supplementary file1 (DOCX 3507 kb) [file 127_2023_2459_MOESM1_ESM.docx]

**Supplementary Information for: Length and associated characteristics of short-term detentions: an analysis of detentions under the Mental Health Act in Scotland, 2006–2018, *Social Psychiatry and Psychiatric Epidemiology***

Moira Connolly^1*^, Lisa Schölin^2^, Gail S Robertson^3^, Arun Chopra^4^

^*^ Corresponding author

^1^ Mental Welfare Commission for Scotland; ORCID: 0000-0002-8421-0165

^2^ Centre for Cardiovascular Science, University of Edinburgh; ORCID: 0000-0002-1348-672x

^3^ University of Edinburgh, School of Mathematics; 0000-0002-2105-3684;

^4^ Mental Welfare Commission for Scotland; ORCID: 0000-0002-5895-0645

**Table S1. Factors associated with STDCs lapsing on day 28 (n=11,289) using a binomial GLMM. Model output is displayed (coefficient estimates, standard error around estimates, z-values and p-values associated with each variable level) and coefficient values and standard errors are given in respect to the 2006 reporting year, gender to females and ethnicity to White - Scottish. Values of Wald chi-square tests comparing models including and excluding each variable and associated p-values are also displayed.**

|  | **Coefficient** | **Std error** | **z-value** | **p-value** | **Wald chi-square** | **df** | **p-value** |
| --- | --- | --- | --- | --- | --- | --- | --- |
| **Year** | - | - | - | - | 114.84 | 12 | *<0.001* |
| 2007 | -0.33 | 0.20 | 1.64 | 0.10 |  |  |  |
| 2008 | -0.01 | 0.19 | -0.064 | 0.96 | - | - | - |
| 2009 | -0.30 | 0.18 | -1.66 | 0.10 | - | - | - |
| 2010 | -0.44 | 0.18 | -2.45 | 0.01 | - | - | - |
| 2011 | -0.62 | 0.17 | -3.68 | <0.001 | - | - | - |
| 2012 | -0.71 | 0.16 | -4.51 | <0.001 | - | - | - |
| 2013 | -0.88 | 0.15 | -5.74 | <0.001 | - | - | - |
| 2014 | -0.75 | 0.15 | -4.90 | <0.001 | - | - | - |
| 2015 | -0.91 | 0.15 | -5.92 | <0.001 | - | - | - |
| 2016 | -1.01 | 0.15 | -6.60 | <0.001 | - | - | - |
| 2017 | -1.01 | 0.15 | -6.52 | <0.001 | - | - | - |
| 2018 | -0.97 | 0.19 | -5.30 | <0.001 | - | - | - |
|  |  |  |  |  |  |  |  |
| **Age** | 2.25 | 0.14 | 16.61 | <0.001 | 275.94 | 1 | *<0.001* |
| **Gender** | - | - | - | - | 0.17 | 1 | 0.68 |
| Female | ref |  |  |  |  |  |  |
| Male | -0.02 | 0.05 | -0.41 | 0.68 |  |  |  |
| **Ethnicity** | - | - | - | - | 13.00 | 6 | *0.04* |
| White Scottish | ref |  |  |  |  |  |  |
| African, Caribbean or Black | -0.36 | 0.22 | -1.65 | 0.10 | - | - | - |
| Other | 0.25 | 0.33 | 0.75 | 0.45 | - | - | - |
| Asian | 0.14 | 0.16 | 0.93 | 0.35 | - | - | - |
| Mixed | 0.46 | 0.47 | 0.99 | 0.32 | - | - | - |
| White - Other | -0.23 | 0.12 | -1.94 | 0.05 | - | - | - |
| White - Other British | -0.21 | 0.10 | -2.08 | 0.04 | - | - | - |

ROC curve AUC = 0.85. Age rescaled by dividing values by 100.

**Table S2. Factors associated with length of revoked STDCs** **(excluding those ending on day 28) (n=7405) using a Poisson-distributed GLMM. Model output is displayed (coefficient estimates, standard error around estimates, z-values and p-values associated with each variable level) and coefficient values and standard errors are given in respect to the 2006 reporting year, gender to females and ethnicity to White - Scottish. Values of Wald chi-square tests comparing models including and excluding each variable and associated p-values are also displayed.**

|  | **Coefficient** | **Std error** | **z-value** | **p-value** | **Wald chi-square** | **df** | **p-value** |
| --- | --- | --- | --- | --- | --- | --- | --- |
| **Year** | - | - | - | - | 97.14.87 | 12 | <0.001 |
| 2006 | ref |  |  |  |  |  |  |
| 2007 | 0.15 | 0.04 | 3.54 | <0.001 | - | - | - |
| 2008 | 0.04 | 0.04 | 0.97 | 0.33 | - | - | - |
| 2009 | 0.03 | 0.04 | 0.84 | 0.40 | - | - | - |
| 2010 | 0.04 | 0.04 | 1.14 | 0.25 | - | - | - |
| 2011 | 0.001 | 0.04 | 0.02 | 0.98 | - | - | - |
| 2012 | -0.06 | 0.04 | -1.73 | 0.08 | - | - | - |
| 2013 | -0.08 | 0.03 | -2.26 | 0.02 | - | - | - |
| 2014 | -0.07 | 0.03 | -2.05 | 0.04 | - | - | - |
| 2015 | -0.09 | 0.03 | -2.72 | 0.007 | - | - | - |
| 2016 | -0.10 | 0.03 | -2.92 | 0.004 | - | - | - |
| 2017 | -0.08 | 0.03 | -2.26 | 0.02 | - | - | - |
| 2018 | -0.10 | 0.04 | -2.64 | 0.008 | - | - | - |
| **Age** | 0.49 | 0.04 | 13.29 | <0.001 | 176.71 | 1 | <0.001 |
| **Gender** | - | - | - | - | 3.72 | 1 | 0.05 |
| Female | ref |  |  |  |  |  |  |
| Male | 0.03 | 0.01 | 1.93 | 0.05 |  |  |  |
| **Ethnicity** | - | - | - | - | 12.01 | 6 | 0.06 |
| White Scottish | ref |  |  |  |  |  |  |
| African, Caribbean or Black | 0.11 | 0.05 | 2.17 | 0.03 | - | - | - |
| Other | 0.07 | 0.09 | 0.85 | 0.40 | - | - | - |
| Asian | 0.04 | 0.04 | 0.93 | 0.35 | - | - | - |
| Mixed | -0.10 | 0.14 | -0.71 | 0.48 | - | - | - |
| White - Other | 0.06 | 0.03 | 1.97 | 0.05 | - | - | - |
| White - Other British | -0.03 | 0.03 | -1.17 | 0.24 | - | - | - |

Age rescaled by dividing values by 100.

**Table S3.** **Factors associated with STDCs progressing to a CTO (n=21,898) using a binomial GLMM. Model output is displayed (coefficient estimates, standard error around estimates, z-values and p-values associated with each variable level) and coefficient values and standard errors are given in respect to the 2006 reporting year, gender to females and ethnicity to White - Scottish. Values of Wald chi-square tests comparing models including and excluding each variable and associated p-values are also displayed.**

|  | **Coefficient** | **Std error** | **z-value** | **p-value** | **Wald chi-square** | **df** | **p-value** |
| --- | --- | --- | --- | --- | --- | --- | --- |
| **Year** | - | - | - | - | 670.40 | 12 | <0.001 |
| 2006 | ref |  |  |  |  |  |  |
| 2007 | 0.18 | 0.13 | 1.34 | 0.18 | - | - | - |
| 2008 | 0.21 | 0.13 | 1.60 | 0.11 | - | - | - |
| 2009 | -0.02 | 0.13 | -0.12 | 0.90 | - | - | - |
| 2010 | 0.06 | 0.12 | 0.50 | 0.62 | - | - | - |
| 2011 | 0.22 | 0.11 | 1.88 | 0.06 | - | - | - |
| 2012 | -0.32 | 0.11 | -2.95 | 0.003 | - | - | - |
| 2013 | -0.35 | 0.11 | -3.30 | <0.001 | - | - | - |
| 2014 | -0.34 | 0.11 | -3.17 | 0.002 | - | - | - |
| 2015 | -0.26 | 0.11 | -2.41 | 0.02 | - | - | - |
| 2016 | -0.44 | 0.11 | -4.19 | <0.001 | - | - | - |
| 2017 | -0.19 | 0.11 | -1.79 | 0.07 | - | - | - |
| 2018 | 1.30 | 0.12 | 11.18 | <0.001 | - | - | - |
| **Age** | 0.64 | 0.09 | 6.85 | <0.001 | 46.91 | 1 | <0.001 |
| **Gender** |  |  |  |  |  |  |  |
| Female | ref | - | - | - | 20.71 | 1 | <0.001 |
| Male | 0.15 | 0.03 | 4.55 | <0.001 |  |  |  |
| **Ethnicity** | - | - | - | - | 60.53 | 6 | <0.001 |
| White Scottish | ref |  |  |  |  |  |  |
| African, Caribbean or Black | 0.11 | 0.14 | 0.75 | 0.45 | - | - | - |
| Other | -0.32 | 0.26 | -1.23 | 0.22 | - | - | - |
| Asian | 0.33 | 0.10 | 3.11 | 0.002 | - | - | - |
| Mixed | 1.24 | 0.27 | 4.67 | <0.001 | - | - | - |
| White - Other | 0.20 | 0.08 | 2.61 | 0.009 | - | - | - |
| White - Other British | 0.34 | 0.07 | 5.09 | <0.001 | - | - | - |

AUC = 0.64. Age rescaled by dividing values by 100.

**Supplementary Figure 1. Median duration of STDCs revoked and lapsed (left) and revoked only (right) by year**

**
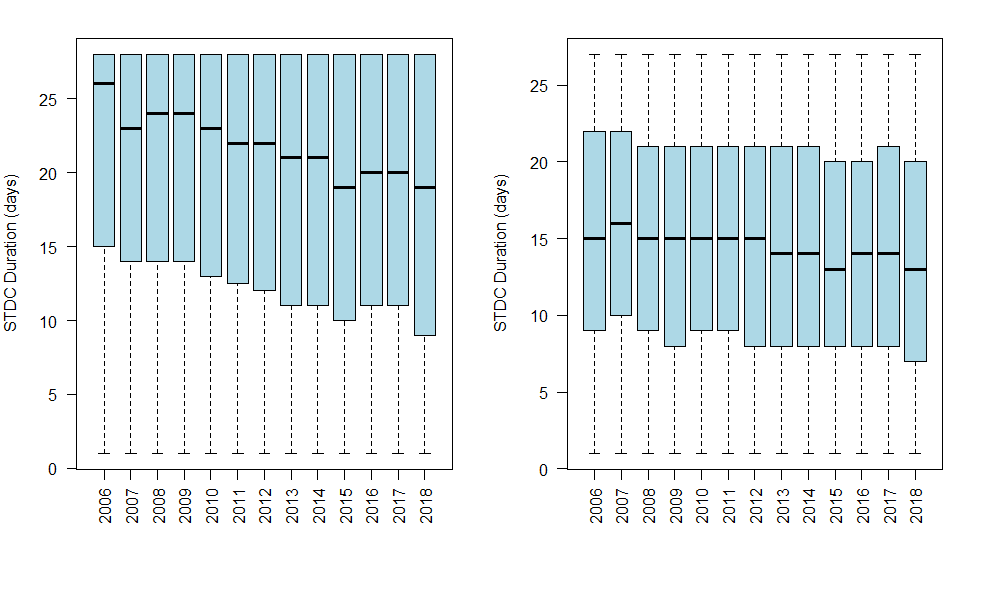
**

**Supplementary Figure 2. Proportion of lapsed (left) and extended (right) STDCs by year**

**
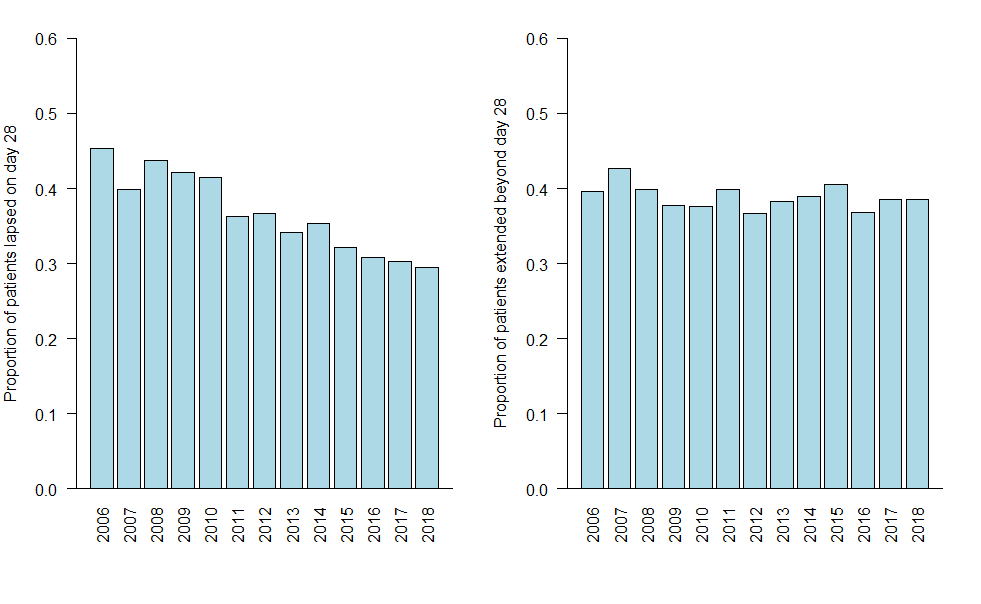
**
